# Socioeconomic factors and adolescent pregnancy outcomes: distinctions between neonatal and post-neonatal deaths?

**DOI:** 10.1186/1471-2458-5-79

**Published:** 2005-07-25

**Authors:** Barry P Markovitz, Rebeka Cook, Louise H Flick, Terry L Leet

**Affiliations:** 1 Saint Louis University School of Public Health, St. Louis, Missouri, USA; 2 Saint Louis University School of Medicine, St. Louis, Missouri, USA; 3 Saint Louis University School of Nursing, St. Louis, Missouri, USA; 4 Washington University School of Medicine, St. Louis, Missouri, USA

## Abstract

**Background:**

Young maternal age has long been associated with higher infant mortality rates, but the role of socioeconomic factors in this association has been controversial. We sought to investigate the relationships between infant mortality (distinguishing neonatal from post-neonatal deaths), socioeconomic status and maternal age in a large, retrospective cohort study.

**Methods:**

We conducted a population-based cohort study using linked birth-death certificate data for Missouri residents during 1997–1999. Infant mortality rates for all singleton births to adolescent women (12–17 years, n = 10,131; 18–19 years, n = 18,954) were compared to those for older women (20–35 years, n = 28,899). Logistic regression was used to estimate adjusted odds ratios (OR) and 95% confidence intervals (CI) for all potential associations.

**Results:**

The risk of infant (OR 1.95, CI 1.54–2.48), neonatal (1.69, 1.24–2.31) and post-neonatal mortality (2.47, 1.70–3.59) were significantly higher for younger adolescent (12–17 years) than older (20–34 years) mothers. After adjusting for race, marital status, age-appropriate education level, parity, smoking status, prenatal care utilization, and poverty status (indicated by participation in WIC, food stamps or Medicaid), the risk of post-neonatal mortality (1.73, 1.14–2.64) but not neonatal mortality (1.43, 0.98–2.08) remained significant for younger adolescent mothers. There were no differences in neonatal or post-neonatal mortality risks for older adolescent (18–19 years) mothers.

**Conclusion:**

Socioeconomic factors may largely explain the increased neonatal mortality risk among younger adolescent mothers but not the increase in post-neonatal mortality risk.

## Background

Adolescent pregnancy has long been considered to increase the likelihood of adverse infant outcomes [1]. The infant mortality rate in the United States (U.S.) for young women has remained persistently high despite widespread study of this phenomenon. In 1999, the infant mortality rate for women under 20 years of age was 10.3 deaths per 1,000 live births compared to 7.0 per 1,000 live births for all ages [2] Although the U.S. teenage (ages 15–19) birth rate has declined substantially during the last decade, from 62.1 to 48.7 per 1,000 women in 2000 [3], this country still leads the developed countries by wide margins. Simple calculations would suggest that, with an estimated 470,000 births to teen mothers in the year 2000, over 1,500 excess infant deaths occurred among adolescent mothers.

The etiology explaining the higher mortality risk for adolescent mothers has been debated for as long as the phenomenon has been observed. Miller argued that socioeconomic status has a direct effect on infant mortality, confounding the role of young maternal age, in a study of 2,019 U.S. counties from 1971–1975 [4]. Many studies point towards low birth weight (including infants born preterm and those small-for-gestational age) as the predominant proximate marker of infant mortality [5-10]. Fraser, in a large study spanning 20 years of live births from Utah, found maternal age to be an independent predictor for poor birth outcomes, but this study did not specifically address infant mortality or socioeconomic status directly [11]. The defining influence of the biology of adolescent pregnancy, risky adolescent behavior, nutrition, race, socioeconomics, and adequacy of prenatal care – or the interaction among these variables – remains controversial. Certainly poverty, minority status, suboptimal prenatal care, poor education and unmarried status are more common in teenage compared to older mothers, and are known risk factors for low birth weight, preterm birth and higher infant mortality in the United States [11]. Adolescent mothers are disproportionately poor. For example, in Missouri for 1997–1999, 79% of births to women aged 14–19 were covered by Medicaid compared to only 35.7% of births to women 20 and older [12]. Among adolescents living in poor communities, 69% report having had intercourse, compared to 37% of those living in more affluent neighborhoods [13]. Support can be found to defend or refute all or none of these factors as independent risk factors [6,7,11,14-16].

We examined linked birth-death certificate data for Missouri residents to assess the role of poverty status and other potential risk factors on infant mortality among teenage mothers. We hypothesized that teen motherhood is highly confounded by poverty, and that there is no independent effect of adolescent age on infant mortality. Our analysis distinguished neonatal and post-neonatal mortality among younger and older adolescent mothers.

## Methods

### Study design

This was a population-based cohort study using Missouri's linked birth-death certificate files for 1997–1999. This period was chosen since it provided a sufficiently large study population with the most current birth-related data. To test the primary hypothesis, we compared infant mortality rates of adolescent and older mothers while controlling for socioeconomic status (defined by participation in poverty assistance programs) and other known and purported risk factors.

### Cohort description

The study population included all singleton live births for mothers who were Missouri residents and less than 20 years of age during 1997–1999. The study population was stratified by maternal age to identify younger (12–17 years) and older (18–19 years) adolescent mothers. A comparison population included a random sample of singleton live births for mothers who were 20–34 years old during 1997–1999, since the infant mortality rates were relatively stable for this age group but higher for older mothers. Women other than non-Hispanic white or African American were excluded, because they comprised less than 3% of all live births in Missouri during the study period.

### Covariates

The following covariates were extracted from the birth certificate: race/ethnicity (non-Hispanic black or non-Hispanic white), maternal education level (recorded as age appropriate based on the number of years of formal education completed by the mother), parity, marital status, tobacco or alcohol use (as dichotomous and categorical covariates based on the number of cigarettes per day or drinks per week), maternal weight gained during pregnancy, adequacy of prenatal care utilization (based on the Kotelchuck index)[17], and socioeconomic status. The poorest women were defined as those receiving food stamps, since this program includes only those with personal incomes less than 130% of the federal poverty level. Poor women were those defined as receiving Medicaid or WIC. In Missouri, pregnant women at or below 185% of the federal poverty level are eligible for Medicaid and WIC. While personal incomes can be used to identify women participating in poverty programs, we cannot assume that all non-participants are above 185% of the federal poverty level. All other women included those who did not participate in any of the three poverty assistance programs. Other covariates were clinical estimate of gestational age (20–28, 29–32, 33–36, ≥37 weeks) and birth weight (<2500, ≥ 2500 grams). Infant mortality included deaths that occurred during the neonatal (0–27 days) and post-neonatal (28–364 days) period. The underlying causes of death were divided into five categories: 1) accidental, 2) respiratory arrest or sudden infant death syndrome, 3) infectious, 4) perinatal or congenital, 5) other deaths.

### Analysis

Chi-square tests and student's unpaired t-tests were used to determine if categorical and continuous covariates, respectively, were significantly different for younger versus older mothers. Overall infant mortality, as well as neonatal mortality and post-neonatal mortality, was assessed as a function of the three age cohorts and stratified by race. Logistic regression was used to assess how each covariate affected the role of maternal age on infant mortality risk. Covariates that altered the odds ratio (OR) for the primary relationship by more than 10% were considered significant and included in the full model. 95% confidence intervals (CI) were computed to estimate the precision of the OR. First-order interaction terms between each covariate and maternal age were tested in a secondary analysis. The significance of adding each interaction was tested with a p-value <0.01. The final model was examined for outliers, influential data and goodness of fit (Omnibus Test and Hosmer and Lemeshow test, p <0.05). The role of each covariate in the full model was also assessed using neonatal and post-neonatal mortality as the primary outcome in separate logistic regression analyses. SPSS (Chicago, IL, version 10.0) was used for all statistical analyses.

This research, reviewed by the Saint Louis University institutional review board, was classified as exempt from the U.S. Department of Health and Human Services regulations for the protection of human subjects. The exemption 45 CFR 46.101(b)(4) permits epidemiologic research that uses existing publicly available data that are maintained in such a manner that subjects cannot be identified directly or through identifiers linked to the subjects.

## Results

The distribution of covariates and outcomes for the three maternal age cohorts is reported in Table 1. Comparing all adolescent mothers (12 to 19 years) to older mothers, newborns of older mothers were, on average, 167 grams heavier than newborns of younger mothers (p < 0.001). Although the younger mothers gained 1.36 kg (3 pounds) on average more during pregnancy than older mothers (p < 0.001), this is not likely to be clinically significant. Compared to older mothers, younger mothers were more likely to be nulliparous (72.6% vs. 29.1%), smoke (27.2% vs. 18.2%), of black race (26.7% vs. 13.8%), and on Medicaid (78% vs. 34.8%), WIC (75.1% vs. 36%), or food stamps (25.5% vs. 16.4%). Not surprisingly, fewer younger mothers were married (20.2% vs. 72.3%) and more had inadequate prenatal care (16.8% vs. 7%). Younger mothers were less likely to have an age-appropriate educational level (72.6% vs. 86.8%), and more likely to have a preterm infant (9.8% vs. 7.6%).

**Table 1:** Characteristics of Missouri women with singleton live births, 1997–1999, by maternal age.

		**12 to 17 (N = 10,131)**		**18 to 19 (N = 18,954)**		**20–35 (N = 28,899)**	
**Characteristic**		**N**	**%**	**N**	**%**	**N**	**%**

Infant mortality		121	1.2	158	0.8	173	0.6
Neonatal mortality		69	0.7	92	0.5	111	0.4
Post-neonatal mortality		52	0.5	65	0.3	60	0.2
Clinical Estimate of Gestational Age	<24 Weeks	40	0.4	50	0.3	59	0.2
	24–28 weeks	102	1.0	110	0.6	139	0.5
	29–32 weeks	161	1.6	252	1.3	260	0.9
	33–36 weeks	808	8.0	1,318	7.0	1,739	6.0
	>36 weeks	8,961	89.0	17,109	90.8	26,571	92.4
Birth Weight Category	Normal (> 2500 gm)	9,114	90.0	17,358	91.6	27,207	94.1
	Low (≤ 2500 gm)	1,017	10.0	1,596	8.4	1,692	5.9
Maternal Race	Missing/other	137	1.4	256	1.4	635	2.2
	Non-Hispanic white	6,688	66.0	14,234	75.1	24,288	84.0
	Non-Hispanic black	3,306	32.6	4,464	23.6	3,976	13.8
Age appropriate education level		8,592	86.1	12,516	66.0	25,086	86.8
Medicaid		7,698	77.1	14,983	80.4	10,070	35.4
W.I.C.		7,761	77.8	14,085	75.6	10,412	36.7
Food Stamp Program		2,060	20.7	5,351	28.7	4,729	16.7
Parity	nulliparous	8,660	85.5	12,375	65.3	8,382	29.0
Unmarried		9,140	90.3	14,064	74.3	7,986	27.7
Tobacco use During Pregnancy		2,486	24.7	5,425	28.7	5,262	18.3
Alcohol use during pregnancy		61	0.6	90	0.5	268	0.9
Kotelchuck index	Unknown	487	4.8	747	3.9	805	2.8
	Inadequate	2,101	20.7	2,789	14.7	2,037	7.0
	Adequate/Intermediate	5,273	52.0	11,121	58.7	18,832	65.2
	Adequate Plus	2,270	22.4	4,297	22.7	7,225	25.0

p ≤ 0.001 for all comparisons

Overall, infants born of the youngest mothers (12 to 17 years) were 1.69 (CI 1.24, 2.31) times more likely to die during the neonatal period and 2.47 (CI 1.70, 3.59) times more likely during the post-neonatal period compared to older mothers Table 2 illustrates the risk of neonatal mortality and post-neonatal mortality for the adolescent age cohorts compared to older mothers, stratified by race. There is an increased risk of infant (9.7 deaths/1000 live births; OR 1.95, CI 1.42, 2.67), neonatal (5.3 deaths/1000 live births; 1.63, CI 1.02, 2.47) and post-neonatal mortality (4.4 deaths/1000 live births; OR 2.58, CI 1.56, 4.24) for the 12–17 year old non-Hispanic white mothers compared to older mothers (5.0, 3.2 and 1.7 infant, neonatal and post-neonatal deaths/1000 live births respectively). Among non-Hispanic white mothers 18–19 years of age, there is an increased risk of infant (6.7 deaths/1000 live births; OR 1.35, CI 1.02, 1.79) and post-neonatal (3.0 deaths/1000 live births; OR 1.75, CI 1.11, 2.75) mortality, but not neonatal mortality (3.7 deaths/1000 live births; OR 1.14, CI 0.79, 1.64). No association between age and infant, neonatal or post-neonatal mortality was seen for non-Hispanic black mothers. However, the crude rates of infant, neonatal and post-neonatal mortality for non-Hispanic black mothers were approximately twice those for non-Hispanic white mothers across all age groups.

**Table 2:** Infant, neonatal and post-neonatal mortality risk by age group, stratified by race

**Race**	**Age group**	**Neonatal mortality**		**Post-neonatal mortality**	
		
		**Rate (per 1000 births)**	**cOR (95% CI)**	**Rate (per 1000 births)**	**cOR (95% CI)**
Non-Hispanic White	12–17 years	5.3	1.63 (1.02, 2.47)	4.4	2.58 (1.56, 4.24)
	18–19 years	3.7	1.14 (0.79, 1.64)	3.0	1.75 (1.11, 2.75)
	20–35 years	3.2	reference	1.7	reference

Non-Hispanic Black	12–17 years	10.4	1.41 (0.84, 2.39)	7.0	1.63 (0.84,3.19)
	18–19 years	9.0	1.23 (0.74, 2.04)	5.0	1.15 (0.59, 2.26)
	20–35 years	7.3	reference	4.3	reference

cOR = crude odds ratio, CI = confidence intervals

Table 3 includes the results of the logistic regression model. Race, appropriate education level, marital status, parity, smoking during pregnancy, level of prenatal care and participation in Medicaid, WIC, or food stamp programs significantly affected the primary effect of maternal age on infant mortality risk and were included in the final model. Controlling for these variables, the risk for neonatal and post-neonatal mortality was 1.43 (CI 0.98, 2.08) and 1.73 (CI 1.14, 2.64), respectively, for the youngest adolescent mothers. There was no increased risk of neonatal or post-neonatal mortality for older adolescent mothers. Furthermore, adjusting for gestational age did not appreciably change any of the adjusted OR for the primary effect of age on infant mortality. First-order interactions between covariates and age, considered as a block, did not reach significance and were dropped from subsequent analyses.

**Table 3:** Logistic regression models: effect of maternal age on infant mortality.

	**Neonatal Mortality**		**Post-Neonatal Mortality**	
**Maternal Age (years)**	**aOR**	**95% CI**	**aOR**	**95% CI**

12–17	1.43	0.98, 2.08	1.73	1.14, 2.64
18–19	1.15	0.83, 1.60	1.04	0.71, 1.53
20–34	1.00	reference	1.00	reference
Non-Hispanic black	2.08	1.56, 2.78	1.45	1.02, 2.04
Age-inappropriate educational Level	1.39	1.01, 1.90	1.73	1.23, 2.44
Unmarried	1.50	1.06, 2.14	1.25	0.83, 1.88
Nulliparous	1.00	0.99, 1.02	1.01	0.99, 1.03
Tobacco Use	1.49	1.10, 2.01	1.26	0.89, 1.79
Kotelchuck Index				
Unknown	4.04	2.53, 6.45	0.88	0.35, 2.21
Inadequate	1.98	1.36, 2.88	1.58	1.05, 2.39
Adequate	1.00	reference	1.00	reference
Adequate Plus	2.89	2.17, 3.85	1.89	1.35, 2.66
Poverty Status				
Poorest *	0.36	0.25, 0.53	2.29	1.36, 3.86
Poor **	0.45	0.33, 0.62	1.60	0.98, 2.61
Other	1.00	reference	1.00	reference

cOR = crude odds ratio, aOR = adjusted odds ratio, CI = confidence intervals

* defined as participation in food stamps

** defined as participation in WIC or Medicaid but not food stamps

Of particular interest was the differential effect of the poverty construct in the model on neonatal and post-neonatal mortality risks. Poverty was defined by participation in Medicaid, WIC and/or food stamp programs, and the effect of such programs appeared to be strongly protective against neonatal mortality when controlling for maternal age, but a significant risk factor for post-neonatal mortality. Mothers participating in the food stamps program (our "poorest" category) had a reduced risk of neonatal mortality (OR 0.36, CI 0.25, 0.53) compared to mothers who did not participate in any of the poverty assistance programs. However, their risk of post-neonatal mortality (OR 2.29, CI 1.36, 3.86) was significantly higher. Stratifying the post-neonatal deaths by cause of death appears to show a higher percentage of deaths due to accidents or infections for the youngest mothers compared to older mothers in our study population (Table 4).

**Table 4:** Underlying causes of post-neonatal death by maternal age (n = 180).

	**Cause of death (%)**					
**Maternal age (years)**	**Accidental n (%)**	**SIDS/respiratory arrest n (%)**	**Perinatal/congenital n (%)**	**Infectious n (%)**	**All other n (%)**	**Total n (%)**

12–17	8(15)	16(31)	12 (23)	4(8)	12 (23)	52 (100)
18–19	11 (17)	34 (52)	9(14)	1(2)	11 (17)	66 (100)
20–35	4(6)	22 (35)	16 (26)	2(3)	18 (29)	62 (100)

chi-square = 14.3, p = 0.08

## Discussion

We conclude that neonatal mortality rates do not differ by maternal age, but post-neonatal rates may be higher for mothers aged 12–17 years after adjusting for socioeconomic and other known risk factors. Our results are most similar to Reichman and Pagnini, who studied all New Jersey births in 1989 and 1990 [18]. They found that controlling for medical and socioeconomic factors accounted for some, but not all the effect of young maternal age on infant mortality. The unadjusted odds ratio of infant mortality among white 15–17 year olds in their study was 2.5; the adjusted value was 1.6. Their study was strengthened by access to linked uniform billing hospital discharge records in addition to state vital statistics. They were therefore able to assess socioeconomic status much more directly in their model, comparing Medicaid recipients and "self-pay" to the privately insured.

In our study, we were limited to inferring poverty status by the dichotomous values of participation in WIC, Medicaid, or food stamps programs as noted on the birth certificate. Recognizing the eligibility criteria for these programs (130% of federal poverty level for food stamps and less than 185% of poverty level for WIC and Medicaid) allowed us to predict confidently income levels of program participants, but we cannot conclude that those who are not participants are of higher income levels. Particularly in the aftermath of the Personal Responsibility and Work Opportunity Reconciliation Act of 1996, many individuals who were eligible may not have enrolled in such programs. Nevertheless, 75–80% of the two younger maternal cohorts were enrolled in Medicaid and/or WIC, compared to only 36% of the older mothers. Such dramatic differences are likely to be truly related to socioeconomic distinctions and not bureaucratic or regulatory obstacles unique to older women. This study is also limited by the validity of the original data entered on birth and death certificates. Of particular concern would be the possibility of differential bias in reporting of information between older and younger mothers since there is evidence that reporting infant mortality may suffer from a racial bias and the race distributions are not identical in our age cohorts [19].

Our finding that participation in WIC and Medicaid appears to be protective against neonatal mortality is consistent with a study by Moss and Carver (1998) [20]. Using data from the 1988 National Maternal and Infant Health Survey, among women whose household income was below 185% of federal poverty level, they found an adjusted odds ratio of infant mortality for WIC participation of 0.64 (CI 0.52, 0.77). In their full model, there was no significant effect of Medicaid, but this compares to an adjusted odds ratio of 1.39 (CI 1.04, 1.86) for "self-pay" individuals versus privately insured as the reference population. Recognizing that WIC and Medicaid, as well as the food stamps program, are not simply markers of low socioeconomic status but are also intervention programs, designed to improve the economic and nutritional condition of the disadvantaged – in particular pregnant women – may explain the apparent protective effect. Our study provides additional evidence that this is a biologically plausible observation. If we accept the premise that low socioeconomic status increases infant mortality by increasing the risk of preterm births, thus resulting in higher neonatal mortality, then adding the infant's gestational age into the logistic regression model should not significantly affect the primary relationship between maternal age and neonatal mortality, and indeed this is the case in our study.

Infant mortality is not a "black box;" the reasons newborns may die are very different from why 11 month olds die. As we can see, distinguishing neonatal from post-neonatal mortality is critical to appreciate fully the relationship between young maternal age and infant mortality. Certainly this increased post-neonatal mortality rate cannot be ascribed to "biologic" factors of younger mothers, where the debate over the mechanism of increased infant mortality with young maternal age has so often raged. The increased risk of post-neonatal mortality among the 12–17 year old mothers remains despite adjustment for covariates in our model suggesting social factors not explained by poverty (at least as measured on our model). It is interesting that the factors protective against neonatal mortality – participation in Medicaid, WIC and/or food stamp programs – are apparent risk factors for post-neonatal mortality (aOR 1.60 for poor and aOR 2.29 for poorest women in the 12–17 year olds). We speculate that whatever protective effect these programs have on neonatal mortality has dissipated after the newborn period, and then they are only markers of lower socioeconomic status. It is intriguing to consider what the causes of death might be in the post-neonatal period and how they differ in younger mothers. Despite our cohort of almost 60,000 infants and an apparent doubling of the accidental and infectious death rates in the youngest mother cohort, these differences did not achieve statistical significance.

## Conclusion

In summary, this population-based cohort study confirms the known association of young maternal age and infant mortality, but adds considerably to our understanding by the distinction of younger from older teens and neonatal from post-neonatal mortality. Socioeconomic factors likely account for most of the young mothers' increased risk of neonatal mortality, but despite adjustment, a substantially increased risk of post-neonatal mortality exists for the youngest mothers. Our analysis points towards an increased risk of accidental and infectious deaths in these infants, raising questions of maternal maturity and ability to supervise adequately these developing infants.

## Competing interests

The author(s) declare they have no competing interests.

## Authors' contributions

The study hypothesis was proposed by LF. All authors shared in the design of this study. RC, BPM and TLL primarily carried out the analysis. BPM and RC drafted the manuscript. All authors read, edited and approved the final manuscript.

## Pre-publication history

The pre-publication history for this paper can be accessed here:


